# Immunomodulatory properties of chicken cathelicidin-2 investigated on an ileal explant culture

**DOI:** 10.1007/s11259-024-10428-7

**Published:** 2024-06-14

**Authors:** Gábor Mátis, Patrik Tráj, Viktória Hanyecz, Máté Mackei, Rege Anna Márton, Júlia Vörösházi, Ágnes Kemény, Zsuzsanna Neogrády, Csilla Sebők

**Affiliations:** 1 Division of Biochemistry, Department of Physiology and Biochemistry, University of Veterinary Medicine, István utca 2., H-1078 Budapest, Hungary; 2 Department of Pharmacology and Pharmacotherapy, Faculty of Medicine, University of Pécs, Szigeti u. 12., H-7624 Pécs, Hungary; 3 Department of Medical Biology, Faculty of Medicine, University of Pécs, Szigeti u. 12., H-7624 Pécs, Hungary

**Keywords:** Antimicrobial peptide, Host defense peptide, Poultry, Gastrointestinal, Cytokines, Inflammation

## Abstract

As the threat posed by antimicrobial resistance grows more crucial, the development of compounds that can replace antibiotics becomes increasingly vital. Chicken cathelicidin-2 (Cath-2) belongs to the group of Host Defense Peptides (HDPs), which could provide a feasible solution for the treatment of gastrointestinal infections in poultry. It is a small peptide produced by the heterophil granulocytes of chickens as part of the innate immune response, and its immunomodulatory activity has already been demonstrated in several cell types. In this study, the effects of Cath-2 on the intestinal immune response were examined using ileal explant cultures isolated from chicken. Regarding our results, Cath-2 displayed a potent anti-inflammatory effect as it alleviated the LTA-caused elevation of interleukin (IL)-6 and IL-2 concentrations, and that of the IFN-γ/IL-10 ratio, furthermore, it increased the concentration of IL-10, alleviating the LTA-evoked decreased level of the anti-inflammatory cytokine. Moreover, when applied alone, it elevated the concentrations of IL-6, CXCLi2, and IL-2, providing evidence of its complex immunomodulatory mechanisms. In summary, Cath-2 was able to modulate the immune response of the intestinal wall not only by reducing pro-inflammatory cytokine release, but also through immune stimulation, demonstrating that it has the ability to improve innate immunity via a complex mechanism that may make it a suitable candidate for the control of intestinal infections.

**Supplementary Information:**

The online version contains supplementary material available at 10.1007/s11259-024-10428-7.

## Introduction

Chickens held in high-density farming environments are at increased risk of infections, especially of gastrointestinal (GI) origin. Enforced growth, crowded housing, and potential heat- or transportation-caused stress can all contribute to the imbalance of the microbiome, which can lead to reduced productivity, weakened immunity, and increased mortality (Kogut 2019; Diaz Carrasco et al. 2019). To minimize the risk of the aforementioned factors, antibiotics have been used in large quantities. However, as antimicrobial resistance has reached a critical level, it poses an excessive threat to human society. Therefore, extensive efforts are being made to reduce the use of antibiotics in the livestock industry (Vidovic and Vidovic 2020). For this reason, the need for antibiotic alternatives to treat GI infections is becoming more and more relevant.

Host defense peptides (HDPs, also known as antimicrobial peptides or AMPs) could provide a feasible solution for the emerging threat of antimicrobial resistance. These small, cationic peptides, produced by the innate immune system of almost all kinds of organisms gained attention for their direct antimicrobial and immunomodulatory activity (Diamond et al. 2009). Although high concentrations are needed to accomplish the former in vivo due to the negatively charged ion and glycosaminoglycan levels (Sun et al. 2018), their immunomodulatory activity can aid in reducing bacterial growth indirectly and in combating harmful inflammatory processes (Diamond et al. 2009).

Having their members isolated from various mammalian and bird species, cathelicidins are considered one of the largest groups of HDPs. They have been proven to possess antibacterial, antiviral, antifungal, and exceptionally high immunomodulatory activity (Agier et al. 2015). In chicken, cathelicidins play a crucial role in the fight against infections as part of the innate immune system. They are expressed in several types of tissues including the GI, respiratory, and urogenital systems, as well as the immune organs including the bone marrow and the bursa of Fabricius (Lee et al. 2016). Chicken cathelicidin-2 (Cath-2) is exclusively produced by heterophils, which are recruited to the site of infection as a key part of the innate immune response (Harmon 1998) and then release the peptide from their granules (van Dijk et al. 2009, 2012; Cuperus et al. 2016a). The advantageous effects of Cath-2 have been described in many species, including chicken, human, rodents, and swine (van Dijk et al. 2016; Coorens et al. 2017b; Kraaij et al. 2017, 2020; Scheenstra et al. 2019; van Harten et al. 2022b; Sebők et al. 2022). It has also been proven to inhibit pathogen-associated molecular pattern (PAMP)-induced pro-inflammatory processes, and to enhance the immune protection of the host helping them to fight infectious diseases (Molhoek et al. 2010; van Dijk et al. 2012; Cuperus et al. 2016b; Schneider et al. 2016; Banaschewski et al. 2017; Verheije et al. 2022; van Harten et al. 2022a).

The present study aimed to investigate the immunomodulatory effects of Cath-2 on the intestinal mucosa of the chicken in vitro, studied on ileal explant cultures exposed to lipoteichoic acid (LTA) for mimicking the inflammatory response. First, the viability of the explants was aimed to be assessed, then the concentration of the following cytokines and chemokines were measured: interleukin (IL)-6, CXCLi2 (also known as chicken IL-8), IL-2, interferon (IFN)-γ, and IL-10, to obtain a comprehensive overview of the enteric innate immune response. The major goal of the study was to contribute to an enhanced understanding of the immunomodulatory effects of Cath-2 in the chicken intestinal wall at the molecular level, which is essential to support the efforts for the future therapeutic use of this HDP in poultry farming.

## Materials and methods

### Explant isolation

The methodology for the isolation of ileal explants followed the previously established protocol developed by our research team (Mátis et al. 2024). A 3-week-old male Ross-308 broiler chicken was sacrificed in accordance with the animal welfare legislation of the European Union, institutional policies, and the guidelines required by the Local Animal Welfare Committee of the University of Veterinary Medicine Budapest. Conforming to Ross Technology guidelines, the chicken’s feeding and handling procedures were constantly monitored. Approval for the experiment was obtained from the Government Office of Zala County, Plant Protection, Food Chain Safety, and Soil Conservation Directorate, Zalaegerszeg, Hungary (approval date: May 11, 2020; license number: GK-419/2020). To maintain the homogeneity of the samples, all of the explants were isolated from a single animal, with the methodology explained below.

All reagents and chemicals utilized were acquired from Merck (Darmstadt, Germany), unless explicitly specified. The chicken was humanely decapitated under CO_2_ anesthesia and following the aseptic opening of the coelomic cavity in a dorsal position, the small intestines were obtained. A 13 cm long ileal segment, positioned 10 cm distally from the Meckel’s diverticulum, was excised, followed by the removal of adipose tissue from its outer surface. Both the inner and outer sides of the section underwent multiple washes with phosphate-buffered saline (PBS) + 1% penicillin-streptomycin solution (Pen-Strep, Gibco [Waltham, MA, USA]), and the longitudinally cut intestine was rinsed until no visible contaminants were observable.

**Fig. 1:**
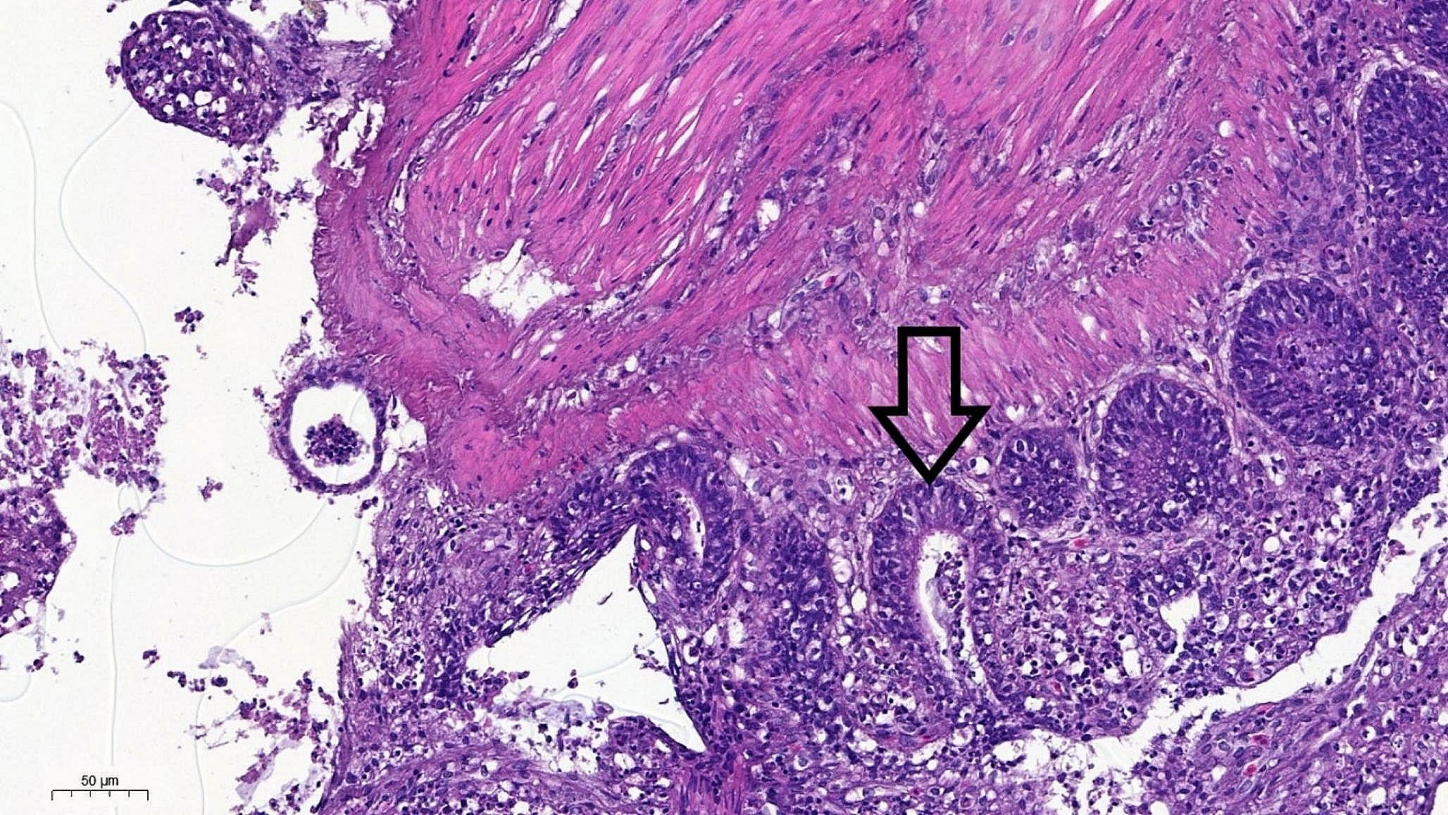
Microscopic image of a hematoxylin and eosin (H&E) stained section of an ileal explant after 14 h of incubation without treatment. The arrow points to the epithelium of an intestinal crypt. Bar line = 50 μm

Explants were extracted with 1.5 mm diameter biopsy punches (MDE [Heidelberg, Germany]), and the freshly gained explants were placed in a 96-well cell culture plate that had been previously coated with type I collagen and filled with 200 µL culture media. The medium employed in the experiment included Dulbecco’s Minimal Essential Medium-F12 supplemented with 2.5% FBS (fetal bovine serum), 1% glutamine, 1% Pen-Strep, and a singular dose of HCMTM SingleQuotsTM Kit (Lonza [Szeged, Hungary]). The latter contained ascorbic acid, bovine serum albumin, hydrocortisone, transferrin, human Epidermal Growth Factor (hEGF), insulin, gentamicin, and amphotericin-B. The explants were incubated at 37 °C under 5% CO_2_ for 2 h. Figure 1 shows a microscopic image of an explant after 14 h of incubation, without any treatment, confirming that the intact micromorphology of the isolated explants was maintained during the entire experiment.

### Treatment of the explants

After the 2-hour incubation, cell culture medium was removed from the wells, and fresh medium substituted with the treatment solutions was added. The treatment solutions included 5, 10, and 25 nmol/mL chicken cathelicidin-2 (Cath-low, Cath-medium, and Cath-high, respectively), 10 µg/mL *Staphylococcus aureus*-derived LTA (LTA), and the combination of the formers (LTA + Cath-low, LTA + Cath-medium, and LTA + Cath-high). Chicken cathelicidin-2 peptide (sequence: RFGRFLRKIRRFRPKVTITIQGSARF-NH2) was purchased from ISCA Biochemicals (Exeter, Devon, UK) in freeze-dried solid form, and LTA from Sigma-Aldrich (Darmstadt, Germany) in lyophilized powder form. Both were dissolved in sterile cell culture medium and diluted to the desired concentrations. Control group received only fresh cell culture medium. Explants were incubated with the solutions for 12 h, thereafter, samples were taken from the cell culture medium and stored at -80 °C until further processing. Treatment groups are explained in Table 1.

**Table 1:** Treatment groups of the study

Treatment group	Cath-2	LTA
Control	-	-
Cath-low	5 nmol/mL	-
Cath-medium	10 nmol/mL	-
Cath-high	25 nmol/mL	-
LTA	-	10 µg/mL
LTA + Cath-low	5 nmol/mL	10 µg/mL
LTA + Cath-medium	10 nmol/mL	10 µg/mL
LTA + Cath-high	25 nmol/mL	10 µg/mL

The first column indicates the name of the groups (also seen in Figures), the second column contains the given concentrations of chicken cathelicidin-2 (Cath-2), and the third column the concentrations of lipoteichoic acid (LTA)

### Measurement of the metabolic activity

The metabolic activity of the explants was assessed with the Cell Counting Kit (CCK)-8 test, which is based on the reduced coenzyme (NADH + H^+^, NADPH + H^+^) production of the cells. The assay was conducted in adherence to the manufacturer’s instructions. After 12 h of incubation and the removal of cell culture medium for sampling, 200 µL fresh medium substituted with 20 µL of CCK-8 reagent was added to each well, then explants were incubated for 2 h under previously described conditions. During incubation, a yellow formazan dye soluble in the culture medium was generated through the reduction of water-soluble tetrazolium salt (WST-8) by cellular dehydrogenase enzymes. The quantity of resultant formazan served as a metric for cellular metabolic activity. Post-incubation, the medium was transferred to a clean 96-well plate, and absorbance was measured at 450 nm using a Multiskan GO 3.2 reader (Thermo Fisher Scientific, Waltham, MA, USA).

### Measurement of extracellular lactate dehydrogenase (LDH) activity

A colorimetric LDH assay kit (Sigma-Aldrich, St. Louis, MO, USA) was used for the quantification of extracellular LDH activity as its elevation is indicative of cell membrane damage. Following the manufacturer’s instructions, components were mixed at room temperature, and a standard dilution series was prepared. A 2 µL sample was measured into a clean 96-well plate and adjusted to 50 µL with the provided buffer solution. The change in absorbance was measured at 450 nm using the Multiskan GO 3.2 reader every minute until the levels of the most concentrated standards were reached. Enzyme activity was computed via the calibration curve outlined in the protocol.

### Measurement of cytokine concentrations

The concentration of CXCLi2 (chicken IL-8) was determined through a chicken-specific sandwich ELISA per the instructions provided by the manufacturer (MyBioSource, San Diego, CA, USA).

The concentrations of IL-2, IL-6, IFN-γ, and IL-10 were measured using the Luminex xMAP method, following the manufacturer’s instructions. 25 µL of samples, standards, controls, and assay buffer were put into a 96-well plate (provided with the kit), then an extra 25 µL of four distinctive colored sets of capture antibody-coated beads were added. After an overnight incubation and the proper washing steps, a mixture of biotinylated detection antibodies and streptavidin phycoerythrin was applied to the plate. Following subsequent incubation periods and a final washing, the plate was filled with MagPix drive fluid, and the beads were resuspended on a plate shaker. Results were read with the Luminex MAGPIX® instrument, and Milliplex Belysa 1.1 software (Merck Millipore, Darmstadt, Germany) was used to collect data. Using bead median fluorescence intensity (MFI) data, the Milliplex Analyst 5.1 software (Merck Millipore, Darmstadt, Germany) produced five-PL regression curves to plot the standard curves for all analytes.

For a more precise assessment of the inflammatory state of the explants, the IFN-γ to IL-10 ratio was calculated. Given their essential roles in regulating inflammatory processes, particularly in the intestinal mucosa where IFN-γ acts as a pro-inflammatory cytokine and IL-10 as an anti-inflammatory counterpart, this ratio provided valuable insights into the dynamics of the inflammatory state (Jamil et al. 2007; Jarry et al. 2008; Ala et al. 2015; Ma et al. 2021).

### Statistical analysis

For the statistical analyses, R version 4.0.3 software was used. Wilcoxon’s signed rank tests were employed for pairwise comparisons as the data showed non-normal distribution using Shapiro-Wilk tests. A significance threshold of *p* < 0.05 was used. Cath-low, Cath-medium, Cath-high and LTA groups were compared with the Control, and LTA + Cath-low, LTA + Cath-medium and LTA-Cath-high with the LTA group. Metabolic activity and LDH activity results were graphically represented as percentages, with the mean of the control group set at 100%. Graphs were generated using GraphPad Prism 9 software (GraphPad Software Inc., San Diego, CA, USA).

## Results

**Fig. 2:**
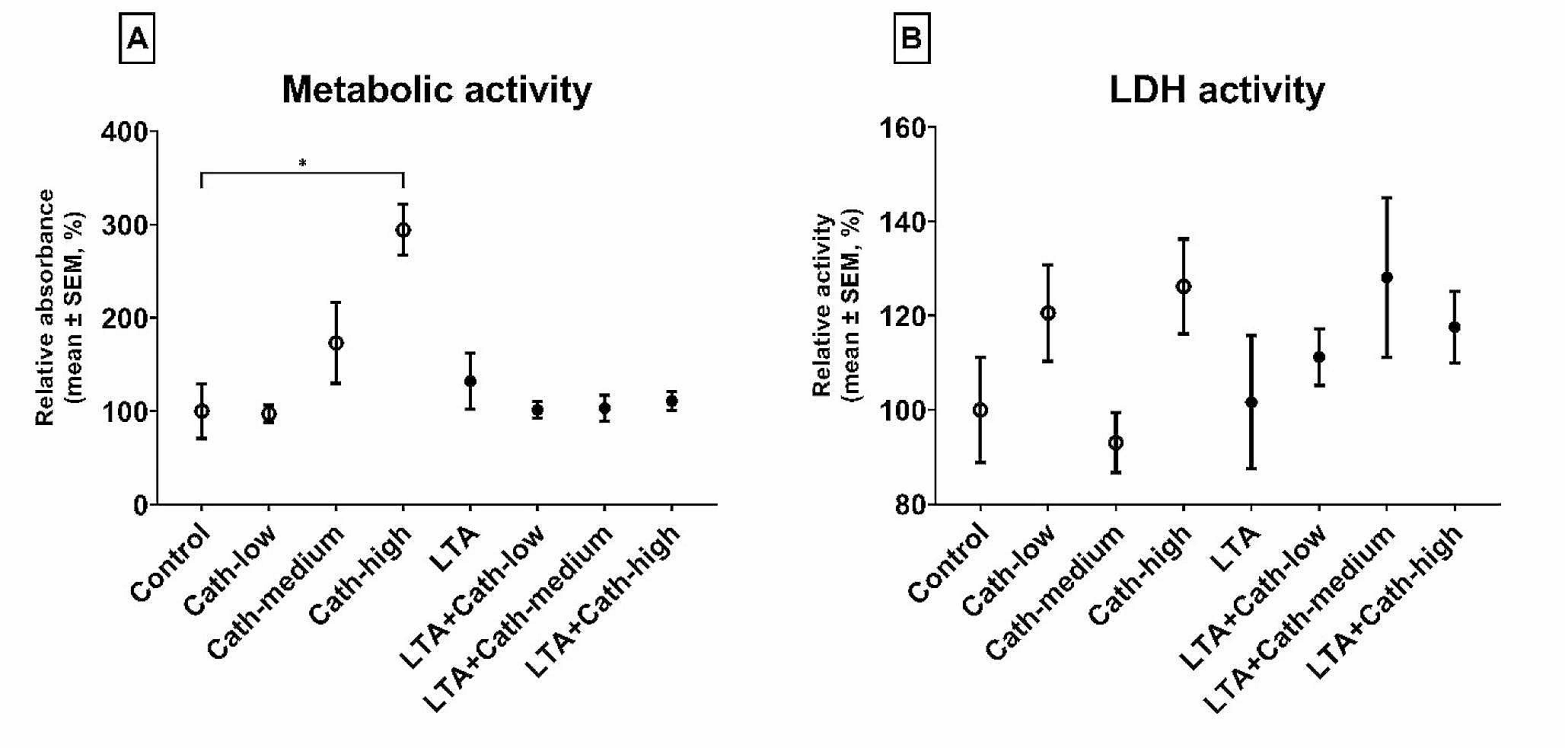
Metabolic activity (**A**) and extracellular lactate dehydrogenase (LDH) activity (**B**) of the explants after treatment. Results are shown in percentage as relative absorbance and activity, where the average of the Control group is considered 100%. Cath-low = 5 nmol/mL of chicken cathelicidin-2, Cath-medium = 10 nmol/mL of chicken cathelicidin-2, Cath-high = 25 nmol/mL of chicken cathelicidin-2, LTA = 10 µg/mL of *Staphylococcus aureus* derived lipoteichoic acid. Control group received none of the treatments. In each group: *n* = 6. *p < 0.05

The metabolic activity of the explants was elevated after treatment with 25 nmol/mL Cath-2 (Cath-high; *p* = 0.010, Fig. 2A). No other treatment influenced the metabolic activity or the LDH activity (Fig. 2B) of the explants in a significant manner.

**Fig. 3:**
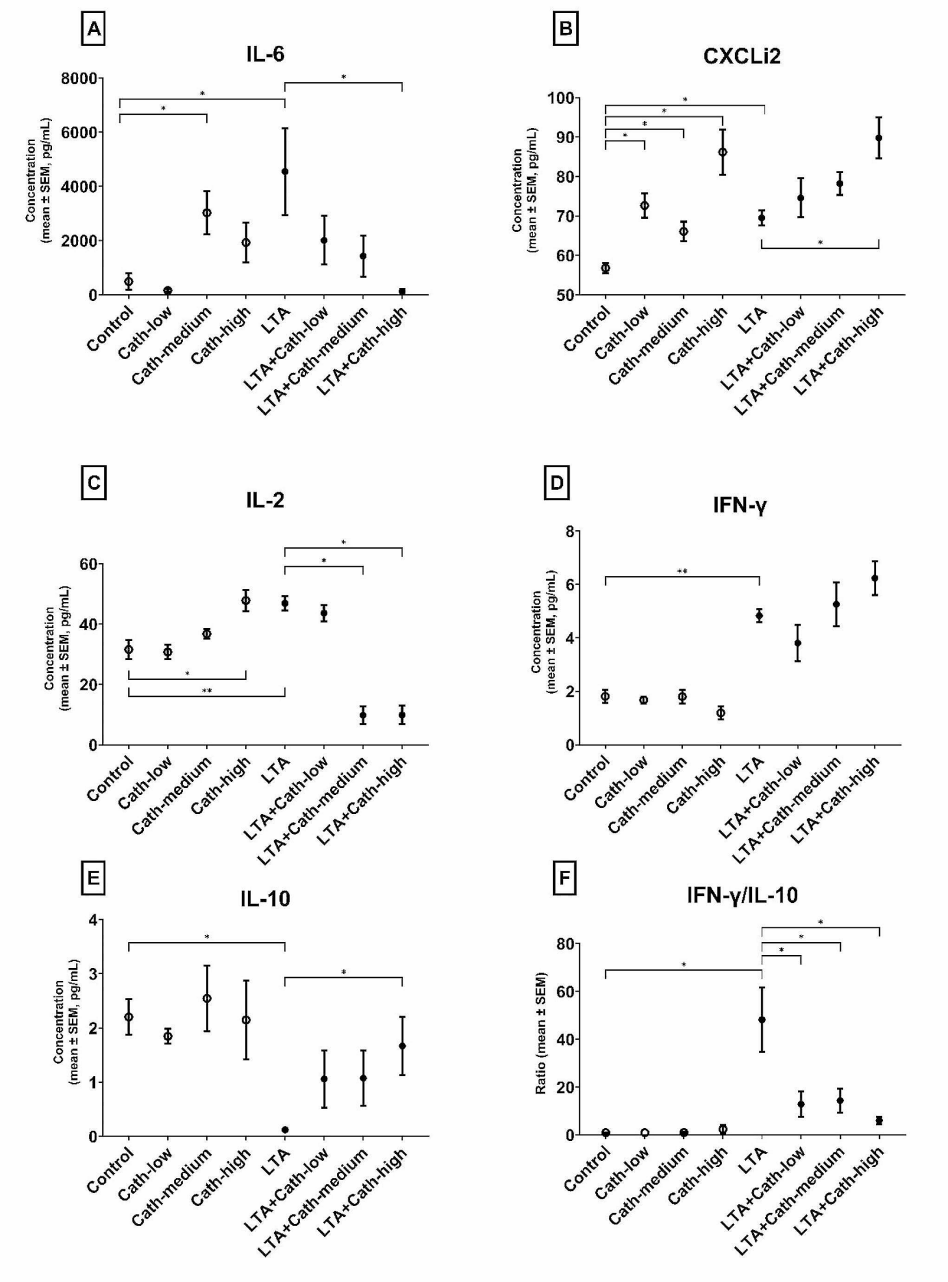
Interleukin (IL)-6 (**A**), CXCLi2 (B), IL-2 (**C**), Interferon (IFN)-γ (**D**) and IL-10 (**E**) concentration, and the ratio of IFN-γ and IL-10 concentrations (**F**) of the cell culture medium after the treatment of the explants. Cath-low = 5 nmol/mL of chicken cathelicidin-2, Cath-medium = 10 nmol/mL of chicken cathelicidin-2, Cath-high = 25 nmol/mL of chicken cathelicidin-2, LTA = 10 µg/mL of *Staphylococcus aureus* derived lipoteichoic acid. Control group received none of the treatments. In each group: *n* = 6. *p < 0.05, **p < 0.01

IL-6 concentration increased following 10 nmol/mL concentration Cath-2 (Cath-medium), and LTA treatment (*p* = 0.016 in both cases, Fig. 3A) compared to Control. The concomitant addition of 25 nmol/mL concentration of Cath-2 to LTA-exposed cells (LTA + Cath-high) decreased cellular IL-6 production compared to the LTA group (*p* = 0.029, Fig. 3A).

CXCLi2 levels were increased after treatment with 5, 10, 25 nmol/mL of Cath-2 (Cath-low, Cath-medium, and Cath-high), and LTA (*p* = 0.019, *p* = 0.027, *p* = 0.014, *p* = 0.016, respectively, Fig. 3B) compared to the Control. Furthermore, the combinatory administration of LTA and 25 nmol/mL Cath-2 (LTA + Cath-high) further increased the concentration of CXCLi2 compared to the already elevated production in the LTA group (*p* = 0.016, Fig. 3B).

25 nmol/mL Cath-2 (Cath-high), and LTA treatment increased the production of IL-2 in comparison with the Control group (*p* = 0.022, *p* = 0.009, respectively, Fig. 3C). Combining LTA with 10 and 25 nmol/mL concentrations of Cath-2 (LTA + Cath-medium and LTA + Cath-high) decreased IL-2 levels compared to the LTA group (*p* = 0.013 in both cases, Fig. 3C).

The concentration of IFN-γ was increased and IL-10 was decreased after LTA treatment (*p* = 0.005, *p* = 0.031, respectively, Fig. 3D and E), which then led to an increase in the ratio of IFN-γ/IL-10 (*p* = 0.036, Fig. 3F). The combinatory exposure to LTA and 25 nmol/mL Cath-2 (LTA + Cath-high) elevated IL-10 levels compared to LTA (*p* = 0.024, Fig. 3E), but had no influence on IFN-γ concentrations. However, the ratio of IFN-γ/IL-10 was decreased by the concomitant application of 5, 10 and 25 nmol/mL of Cath-2 (LTA + Cath-low, LTA + Cath-medium, and LTA + Cath-high) compared to the group receiving only LTA (*p* = 0.048, *p* = 0.024, *p* = 0.028, respectively, Fig. 3F).

## Discussion

In chickens, cathelicidins play a crucial role in the fight against infections as part of the innate immune system. They are expressed in several types of tissues including the GI, respiratory, and urogenital systems, as well as the immune organs including the bone marrow and the bursa of Fabricius (Lee et al. 2016). Cath-2 is exclusively released from the granules of heterophils, which are recruited to the site of infection as a key part of the early immune response (Harmon 1998; van Dijk et al. 2009, 2012; Cuperus et al. 2016a).

It is without doubt that the role of Cath-2 in the immune response is essential; however, the medical application of the peptide may face some specific obstacles. For example, some HPDs may potentially exhibit cytotoxic effects towards host cells, and although Cath-2 is generally considered safe in this regard, there are studies indicating a decrease in cell viability. For instance, it reduced the metabolic activity in a chicken macrophage cell line at 40 nmol/mL concentration (van Dijk et al. 2016), and also in a chicken hepatic co-culture while decreasing the extracellular LDH activity at 10 nmol/mL concentration (Sebők et al. 2022). Nonetheless, this effect did not occur with the chicken ileal explants used in this study, as neither the metabolic activity, nor the LDH activity was altered by 5 and 10 nmol/mL concentrations of Cath-2 which might imply the applicability of Cath-2 for gastrointestinal diseases. Although, it is worth considering, that the highest concentration of Cath-2 treatment (25 nmol/mL) significantly increased the metabolic activity of the cells, by approximately 200%.

The increased metabolic activity can not only be caused by the production of NADH + H^+^, as the CCK-8 test utilized in this study also measures the cellular production of NADPH + H^+^, which is known to have a diverse role in the regulation of inflammatory mechanisms (Ting et al. 2023). For example, NAPDH + H^+^ is required for fatty acid synthesis, the upregulation of which is characteristic of inflammatory processes. Fatty acids are necessary to produce pro-inflammatory lipid mediators (like prostaglandin E_2_) (Everts et al. 2014) and have an important role in the organization of lipid rafts (Carroll et al. 2018), and in the regulation of phagocytosis due to the need of membrane formation (Gagnon et al. 2002; Ecker et al. 2010). The Cath-2-associated increased production of NAPDH + H^+^ aligns with our results indicating the pro-inflammatory effects of the same concentration of Cath-2 as it elevated the IL-2 and CXCLi2 levels as well.

Activation of the immune response to enhance antimicrobial activity is an important function of cathelicidins and has been established by several previous studies. For example, it has been proven that the human cathelicidin LL-37, as well as Cath-2, activates the NLRP-3 (NOD-, LRR- and pyrin domain-containing protein 3) inflammasome, which leads to the caspase-1-dependent release of pro-inflammatory cytokines (Kahlenberg et al. 2013; Peng et al. 2022b). In the present study, the sole application of Cath-2 increased the levels of CXCLi2, IL-6 and IL-2, and the cellular CXCLi2 release was stimulated by 25 nmol/mL Cath-2, even under LTA-provoked inflammation. These findings are in line with other results obtained from the research of Cath-2 (and Cath-2 derived peptides), where it has been able to induce the production of different cytokines and chemokines (e.g. IL-8, RANTES, IL-1β and IL-1α) (van Dijk et al. 2016; Peng et al. 2022a, b; Sebők et al. 2022).

IL-2 has an especially important role in gut homeostasis as it enhances immune tolerance and prevents chronic inflammation of the intestinal mucosa by adjusting the function of regulatory T-cells (Sadlack et al. 1993; Malek 2008; Boyman and Sprent 2012; Zhou et al. 2019). It is mainly produced by activated T-cells during inflammation and has both pro- and anti-inflammatory functions (Bachmann and Oxenius 2007). In this study, both, Cath-2 and LTA applied alone elevated the levels of this cytokine, while Cath-2 lowered the LTA-caused increase, indicating a protective and anti-inflammatory effect of this HDP. However, to further evaluate the protective function of Cath-2 in the immune system of the intestines, additional research is needed regarding the distribution of different T-cell populations, the lack of which is a limitation of this study.

The pro-inflammatory effects of LTA on chicken ileal explant cultures and chicken hepatic cell cultures have been investigated previously by our research group, where LTA was shown to be a suitable tool to stimulate inflammatory responses (Sebők et al. 2021, 2022, 2023; Mátis et al. 2024). Based on our present results, Cath-2 had mostly anti-inflammatory effects under LTA-evoked inflammatory conditions as it successfully alleviated the LTA-triggered elevation of IL-2 and IL-6 levels, as well as IFN-γ/IL-10 ratio. Similar results were obtained from previous studies where the anti-inflammatory properties of this peptide and its derivates were investigated. For example, they have been proven to neutralize the LPS and LTA-induced release of pro-inflammatory cytokines (Molhoek et al. 2010; Coorens et al. 2017b; Sebők et al. 2022) and to inhibit *Pseudomonas aeruginosa*-caused macrophage activation (Coorens et al. 2017a).

Furthermore, in this study, LTA decreased the concentration of the anti-inflammatory IL-10, and Cath-2 raised it back to the level observed in the Control group. Moreover, the production of IFN-γ was elevated after treatment with LTA, but Cath-2 did not affect its level. Despite this, the LTA-caused elevation in the IFN-γ/IL-10 ratio was lowered by all concentrations of this HDP, suggesting an explicit anti-inflammatory mechanism. The ratio of these two cytokines is especially useful in studies involving the intestinal immune system as they both play a particularly important role in its regulation (Tau and Rothman 1999; Jarry et al. 2008; Izcue et al. 2009; Ala et al. 2015; Ma et al. 2021). In the recent study, the anti-inflammatory effects of Cath-2 were the most prominent at 25 nmol/mL concentration as it was the only one decreasing IL-2 and IL-6 concentrations, the IFN-γ/IL-10 ratio, and elevating the IL-10 levels compared to the group receiving only LTA.

In conclusion, our present results provide important novel data on the immunomodulatory effects of Cath-2 on the small intestinal mucosa of the chicken. Based on the data gained, it can be stated that Cath-2 has a broad anti-inflammatory effect on chicken ileal explants as reflected by the alleviation of the LTA-triggered pro-inflammatory cytokine release, mostly at higher concentrations, without being cytotoxic. Moreover, the immunostimulant action of this HDP could also be observed, predominantly when administered to non-inflamed cells, which can prove to be an efficient mechanism to provide protection against infections. Therefore, Cath-2 can be considered a suitable candidate for the treatment of bacterial diseases by maintaining the appropriate inflammatory homeostasis and with further in vivo studies may significantly contribute to the reduction of antibiotic use for enteric infections.

### Electronic supplementary material

Below is the link to the electronic supplementary material.

Supplementary Material 1

## Data Availability

Data is provided within the Supplementary information file 1.
